# Flexible, Stretchable Sensors for Wearable Health Monitoring: Sensing Mechanisms, Materials, Fabrication Strategies and Features

**DOI:** 10.3390/s18020645

**Published:** 2018-02-22

**Authors:** Yan Liu, Hai Wang, Wei Zhao, Min Zhang, Hongbo Qin, Yongqiang Xie

**Affiliations:** 1 Key Laboratory of Electronic Equipment Structure Design, Ministry of Education, Xidian University, Xi’an 710071, China; liuy@xidian.edu.cn (Y.L.); qhb0920qhb@xidian.edu.cn (H.Q.); yqxie@xidian.edu.cn (Y.X.); 2 School of Aerospace Science and Technology, Xidian University, Xi’an 710071, China; wanghai@mail.xidian.edu.cn (H.W.); minzhanghk@gmail.com (M.Z.)

**Keywords:** wearable health monitoring, sensors, flexibility and stretchability

## Abstract

Wearable health monitoring systems have gained considerable interest in recent years owing to their tremendous promise for personal portable health watching and remote medical practices. The sensors with excellent flexibility and stretchability are crucial components that can provide health monitoring systems with the capability of continuously tracking physiological signals of human body without conspicuous uncomfortableness and invasiveness. The signals acquired by these sensors, such as body motion, heart rate, breath, skin temperature and metabolism parameter, are closely associated with personal health conditions. This review attempts to summarize the recent progress in flexible and stretchable sensors, concerning the detected health indicators, sensing mechanisms, functional materials, fabrication strategies, basic and desired features. The potential challenges and future perspectives of wearable health monitoring system are also briefly discussed.

## 1. Introduction

Human beings have been fighting against the health issues for millions of years. The emergence of disease often elicits great anxiety and pain to people. In order to treat the diseases and mitigate the worry about sudden illness, work is steadily progressing in evaluating the body conditions from the ways based on personal experience to simple ancillary equipment, and then to systematic medical theory and powerful examining armamentariums. Nowadays, accurately acquiring real-time health signals has been achieved in medical institutions, which helps a lot in diagnosing disease and selecting proper medical measures. However, most medical are highly concentrated in central hospitals, making healthcare services laborious and time-consuming when people gather there in large numbers. Patients, especially the ones in the developing areas, may suffer more pain and even death for the lack of timely and effective treatment. Moreover, the high cost of purchasing, using and maintaining these medical facilities also brings heavy economic burden to hospital and patients, which can further hinder the diagnosis and treatment of diseases.

To implement healthcare service in a convenience and low cost way, many household medical appliances have been developed with the advent of minimized devices and paper-based analytical tools, such as electronic sphygmomanometer, portable electrocardiogram (ECG) monitor and glucometer. These devices can match the assured requirements of WHO in medical devices (affordable, sensitive, specific, user friendly, robust and rapid, equipment free and deliverable) [1], but are still not good enough. Main limitation of these traditional household devices is not wearable and thus cannot be continuously used in daily life. Furthermore, advancements of Internet of things (IoT) in medical service also require high-performance sensing devices with the capability of multi-functionality and real-time detecting [2]. Obviously, incorporating high-level wearability into household medical devices is urgently required to realize portable, remote and real-time health monitoring system. However, the wearability here is not just referring to installing the devices onto human body, like implanting heart pacemaker or catheter, which could cause infections and secondary damage for their invasiveness and uncomfortableness.

To bestow clinical diagnoses with wearable, noninvasive, comfortable and efficient health monitoring system, a new concept for decentralized medical service is proposed based on flexible/stretchable sensors, which act as the collector of physiological information in wearable health monitoring appliances. In the past few years, various configurations for wearable sensors have been developed and demonstrated their capability in monitoring human body conditions [3,4], including heart and breath rate [5,6,7], wrist pulse [8], facial expression [9] and vocalization [10,11,12], metabolism [13,14,15,16,17], etc. However, the development and commercialization of wearable sensing devices are still at a relatively slow speed. This status can be attributed to the following aspects. Firstly, the features relevant to the comfort of long-term wearing should be taken into account, including bio-compatibility, compactness with skin, durability, size and weight [18]. Therefore, ordinary constructing methods for semiconductor devices are no longer suitable. Second, human body possesses very complex attributes, and many different stimuli may be simultaneously loaded to the sensing devices during the monitoring. Thus, the capability of differentiating target and nontarget parameters is required. Moreover, adequate attention should be paid to the strategies for signal delivery and power supply to simplify the system. Despite the abovementioned challenges, a continuous evolution of wearable sensors has been taking place throughout the years, motivated by the emerging breakthroughs in material science, nanotechnology and fabrication techniques.

**Figure 1:**
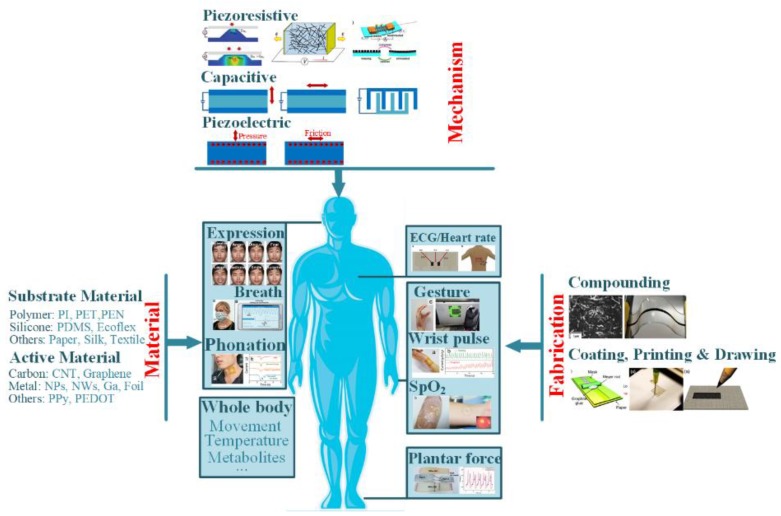
Illustration of the basic concerns of wearable sensors for health monitoring.

Recently, several good overviews have been reported on the advanced achievements in wearable electronics. These works presented in-depth view on various devices (e.g., physical sensors [19], electrochemical sensors [20,21,22], power source devices [23,24], interconnects [25,26] etc.), available materials (e.g., paper [27,28,29], silk fibroin [30] for substrate, and CNT, graphene, metal nanowires for functional element) and fabrication techniques (handwriting or drawing [31,32], printing [33,34] etc.). However, many reviews mainly focus on one aspect of wearable sensors, and a systematic has not been well expressed. This review thus proposes a comprehensive review of the flexible/stretchable sensors and their applications in human health monitoring. The concerned items include detectable indicators, sensing mechanisms, functional materials, fabrication strategies and demanded features. The basic concerns can be found in Figure 1, and the referred contents will be presented in the following sections. Challenges and promising directions for practical researches will also be briefly discussed.

## 2. Detectable Indicators in Health Monitoring

The health status of human body can be indicated by a variety of physiological parameters, and their individual roles and interactions with others require special attentions when evaluating and diagnosing a certain disease. Roughly, the detectable indicators in health evaluation are categorized as: (1) body motions, including hand, limb, foot, face, throat etc., (2) vital signs, including breath/heart rate, wrist pulse, ECG, blood pressure, skin temperature, SpO_2_, etc., and (3) metabolism parameters, including glucose, pH, electrolytes, lactic acid, etc. These parameters have been detected by various wearable sensors (as shown in Table 1), which can be regarded as physical and chemical ones on the basis of their detecting targets. The physical devices, inspired by the pioneering studies of John A. Rogers, Takao Someya and Zhenan Bao, mainly measure the physical-parameter-based indicators like body motions, breath, heart beating, skin temperature, and electrophysiological signals. The latter ones, usually electrochemical biosensors, deal with the metabolism parameters. The detailed descriptions will be covered below.

### 2.1. Body Motions

#### 2.1.1. Hand and Limb

Hand and limb are the most active part in our body, and their motions create most of human activities in daily life. Complete implementation of these movements can guarantee the high-quality life and high-efficiency work for us. Meantime, hand gesture also works as a sign langue to transfer information, which is often used by the people with langue problems and applied in human–machine interface (HMI) devices [35].

Fingers are the most important part in hand, and their motions participate in nearly every function of hand. Flexible or stretchable strips are often used to monitor fingers, which can be individually worn on the target fingers or integrated into a glove. The movement of fingers can change the bending/stretching state of sensing strips, and then induce a finger-curvature-related variation to their electrical parameters. The obtained signals of fingers can be used to monitor personal habit in work and daily life, diagnose and prognosis of Parkinson’s disease, translate sign langue [36] and transfer commands to machine through available interface [37]. In addition to bending motions, limbs can also twist with the help of joints in shoulder, elbow, wrist, coxae, knee and ankle, thus larger but more exquisite strips are required to monitoring their motions.

**Figure 2:**
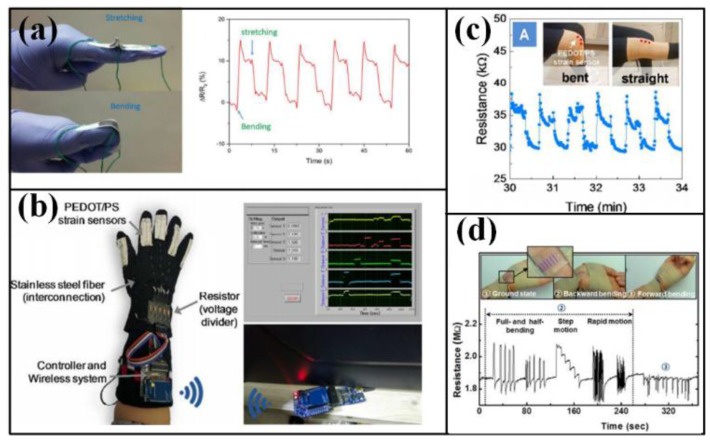
Movement detection of fingers, wrist and knee: (**a**)detection of finger movement by single strip; (**b**) detection of finger movement by glove-like device; (**c**) detection of knee and wrist movement. Reproduced from [38] with permission of The Royal Society of Chemistry. Reprinted with permission from [36]. Copyright 2107 American Chemical Society. Reprinted with permission from [49], Copyright (2015) American Chemical Society.

Many efforts have been invested to detect the movement of hands and limbs, especially fingers. The simplest way to observing finger is adhering a strip to the target one, and the strip can be made of paper with pencil trace or organics membrane with carbon or metals [38,39] (as shown in Figure 2a). However, the adhesion may bring uncomfortableness to the covered skin, and neatening the electrical wires will also become a chore if more strips are adhered. To promote the wearability, glove-like devices are proposed. The sensing strips are integrated in the finger parts, and the remaining area can be the holder for the electrical wires and connectors, which offers an easier and comfortable way to wear the device (as shown in Figure 2b) [35,36]. With the help of these detectors, abundant motions realized by fingers have been identified, such as clicking or wheeling a computer mouse [39], making a fist [40], presenting a gesture [3]. The validated motions can be further transferred into a machine langue under certain rules to input characters or numbers [35,36,41], control robotic arms [37,42], translate Morse code [34], etc. As for limbs, the strips are usually mounted around the joints to detect the bending and straightening processes (as shown in Figure 2c) [36,40], and the acquired signals can be used to identify human motion patterns [43,44,45], analyze body gesture and drive posture games [46,47,48]. The motions of hand and limb can also be indicated by the tension and relaxation of relative muscles, but more sensitive devices are required to fulfill the referred measurements [7,49,50]. Wearing an accelerometer-based device also helps a lot in human motion monitoring, but more sophisticated scheme is needed to realize the device flexibility and stretchability [51,52].

#### 2.1.2. Plantar

Foot stands the largest pressure for the longest time among our body, and thus measurement and analysis of plantar pressure is crucial in many healthcare applications, including clinical gait analysis, foot-related diseases postoperative evaluation, interactive HMI in lower limb rehabilitation and performance monitoring and injury prevention for athletes [53,54,55,56,57]. Commercialized plantar pressure measuring devices have been well studied and taken into markets for many years. Relative products can be categorized as: (1) plate devices, such as the Sports Balance Analyzer™ and Footprint Plus™ by Tekscan [58,59], Emed^®^-systems by Novel [60] and Footscan^®^ system by RSscan [61], (2) in-shoe devices, e.g., F-Scan™ system by Teksacn [62] and Pedar^®^ system by Novel [63]. These devices feature high precision, excellent reliability and mature evaluation system. Meanwhile, these mature solutions cannot be afforded by ordinary persons for daily usage, and are quite apart from the lack of wearability. Thus, the research on wearable plantar pressure measurement mainly focuses on the devices with simple structure, low price and potential for large-scale application in common families. To realize wearability, the in-shoe scheme is utilized to ensure mobility and the soft materials are used to construct the flexible sensing units. Different sensing units have been incorporated into shoes, including embedded commercial/tailored air pressure sensor [64,65], force/pressure sensor [57,66], piezoelectric polymer film [67,68,69], printed interdigitated capacitor [70], etc. These sensing units might be arranged into an array to cover the whole plantar area, in which a set number of sensors will be aligned under a certain rule, and necessary electrodes and wires should be well designed to ensure the signal and power transferring. Meantime, arranging fewer sensors in specific areas, such as hell, midfoot, metatarsal and great toe, can also accomplish the measuring work. According to the anatomical partition of foot sole, at least 15 sensing units are needed to completely represent the plantar pressure [54]. Compared with the devices for hand and limb, the ones for measuring plantar pressure require much larger range, better abrasion resistance and longer service life to fulfill the arduous and long-term measurement.

#### 2.1.3. Face and Throat

**Figure 3:**
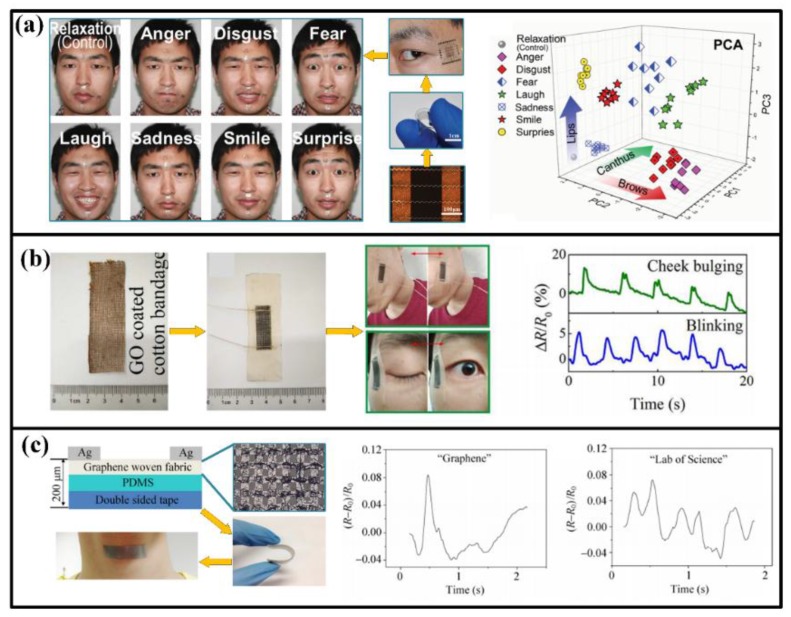
Detected indicators in face and throat: (**a**) Facial expression; (**b**) blinking and cheek bulging; (**c**) vocalization of “graphene” and” lab of science”. Reproduced from [9] with permission of WILEY. Adapted with permission from [72], Copyright (2017) American Chemical Society. Adapted with permission from [10], Copyright (2015) Springer.

Facile expression is a main way to show personal emotion. When the body status changes, expression will also show an evident variation (maybe from relaxation to suffering). Essentially, the expression is driven by the movement of facile muscles, which can be detected by highly sensitive strain sensors and identified by big data systems, as shown in Figure 3a. The tape- or tattoo- like sensors are usually attached on the skins in the forehead, canthus, philtrum, angulus oris and chin, which contains the basic muscle groups for most expressions [71]. After obtaining the muscle-movement-related signals, expressions of eight emotions (anger, disgust, fear, laughter, sadness, smiling, surprise, and relaxation) can be classified and identified through principal component and analyses [9]. Also, some other facile motions like blink and mastication can also be detected, as shown in Figure 3b [7,72].

Many people cannot speak out with sound languages owing to acquired diseases or accidents, but their throat muscles may still move when they attempt to vocalize. Therefore, collecting and identifying information through the stretching and shrinking of throat muscles can be a favorable method to let these people “speak” again. For human, different vocalizations correspond to different muscle strained conditions, and the attached strain sensors will be stretched or compressed along with the muscles, thereby altering their electrical parameters (e.g., resistance or capacitance) and then revealing the information. In order to distinguish the minimum structural unit of a word, the strain sensors should be extremely sensitive to the feeble muscular motions when speaking a letter or word, no matter with or without vocalization [10]. With the help of recently designed devices, many language information has been acquired, including English letters, words, phrases and Chinses characters [4,10,11,73,74]. Moreover, the detected muscle motions can be converted into mechanical vibration and further into controllable and predesigned sounds with the help of intelligent artificial throat, which will be a significant assist for the people with acquired vocalization problems [12,75]. Meantime, muscle also exhibit strains in deglutition, which can be detected for medical purposes [72].

### 2.2. Vital Signs

Important vital signs of patients should be persistently monitored in intensive care units. Many of them directly indicate the outbreak of serious diseases like heart attack, hypertension and asphyxia. Thus, real-timely monitoring these signs are very important in the nursing of high-risk groups. Nowadays, the detection of many vital signs has been revolutionized by the progress of wearable sensing electronics.

#### 2.2.1. Breath

**Figure 4:**
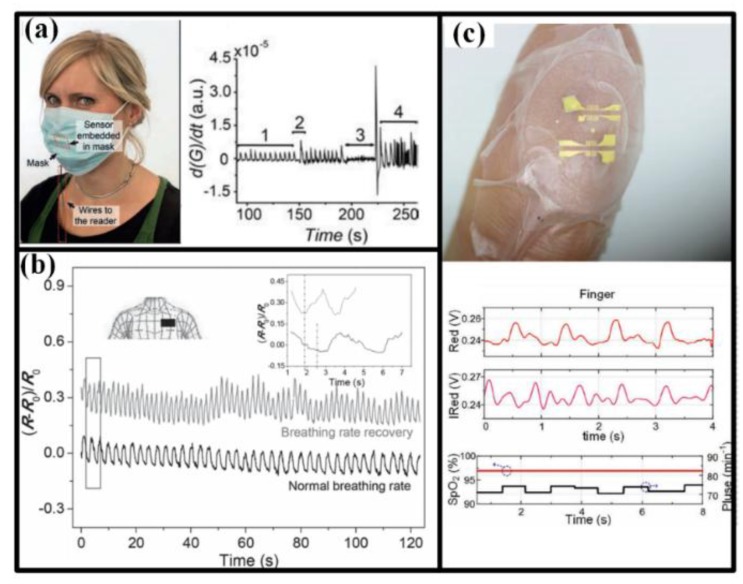
Detection of breath and SpO_2_: (**a**) Detecting breath with a sensor embedded in mask, and the curve shows the results under normally breathing (1), taking a deep breath (2), paused (3) and randomly breathing (4); (**b**) Detecting breath with a strip sensor worn in the chest; (**c**) Detecting the SpO_2_ with a sensor attached on fingertip. Reproduced from [5] with permission of WILEY. Reproduced from [7] with permission of WILEY. Reproduced from [79] with permission of WILEY.

Breath is the only way for body to get oxygen, which is the first important matter for human life. Unusual respiration pattern is a critical symptom for many disorders such as sleep apnea, asthma, chronic obstructive pulmonary disease, and anemia. The breath can be sensed from nostrils or mouth by the airflow and form chest or abdomen by the cavity volume variation, as seen in Figure 4. For nostrils or mouth, a face mask is often used to install the sensors, and the breath rate and intensity can be measured from the airflow-induced strain [73] and the transient difference of moisture in inhaled and exhaled air [5,76,77,78]. For chest/abdomen, volume variation of cavity can generate an obvious apophysis in our body, inducing a periodic motion that can be detected by the wearable strain sensors [7,74]. Meantime, the breath efficiency can be evaluated by SpO_2_, whose measurement has been accomplished by the flexible optoelectronic device mounted on the tip of fingers (Figure 4c) [79].

#### 2.2.2. Heart Rate, Pulse and ECG

Heart is the power source of hemokinesis, and any decline in heart function will lead to significant effect on human health. Therefore, exhaustive inspecting and monitoring of heart working state are of prime importance in healthcare services. Beating rate and pulse are the simplest indictors for heart, and they can be detected by the strain, pressure or acceleration sensors in chest, wrist, neck or finger tips [7,8,74,80,81]. The radial artery pulse waveform can be further processed to get the blood pressure [8], and feeling the wrist pulse is also a diagnostic method in traditional Chinese medicine. ECG can provide detailed information about the beating process of heart, which is very helpful in predicting serious heart attacks. Electrodes is the key element for acquiring ECG, and conventional ones are usually attached in thorax, wrist and ankle. To extend the application of ECG monitoring, lots of improved electrodes are proposed. It was reported that the pencil lead based electrodes could acquire better ECG signals than normal Ag/AgCl ones under freshwater and saltwater conditions [6]; the liquid metal ink provided the ECG electrodes with drawability and simplified installation [82]; along with other biosensors, the bipolar ECG sensor could be co-printed onto a flexible substrate together with other detectors to yield more comprehensive understanding of human health [13,83].

#### 2.2.3. Skin Temperature

Precisely measuring skin temperature can, together with other vital signs, provide clinically relevant information about human physiology, including cardiovascular health, cognitive state and malignancy, etc. Traditional thermometry methods use sophisticated infrared digital cameras or paste-on temperature sensors for single-point measurements, which cannot realize the continuous and cost-effective temperature mapping, which is demanded by many clinical applications. Therefore, the tattoo with array of sensing units, like p-n junctions, thermocouples and thermal resistors, can be an ideal candidate for high-quality thermometry [84,85,86,87].

Though many vital signs have been detected by wearable devices, simultaneously detecting all these indicators by using only few sensors is still a challenge. Aiming at constructing high-performance physiological measurement electronics, Prof. John A. Rogers’ group presented an epidermal electronic system, by which the electrical activity produced by heart, brain, and skeletal muscles were measured in conformal, skin-mounted modes without conductive gels or penetrating needles [88]. The obtained signals could be used to differentiate the modes of vocalization and body motion, and then fulfilled the HMI function to transfer information and control the machine. In addition, the biocompatible tattoo electronics were mechanically invisible, and could be continuously worn for up to 24 h or more. Meanwhile, the devices durability in the challenging areas, such as the elbow, might not be maintained because of the repeated full-range movements.

### 2.3. Metabolism Parameters

**Figure 5:**
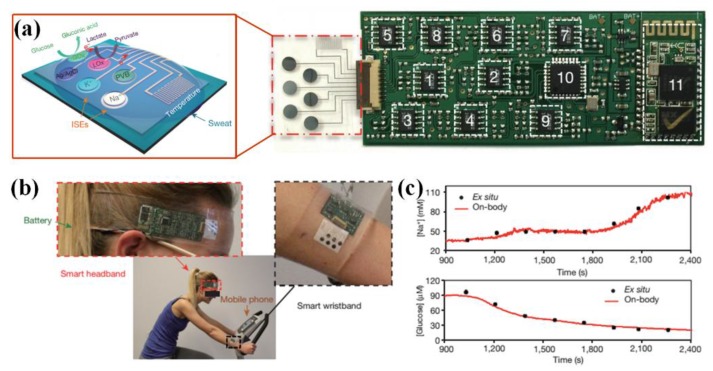
Detection of metabolism parameters: (**a**) the sensor array for simultaneously and selectively measuring the Na^+^, K^+^, glucose and lactate in sweat; (**b**) the devices worn in forehead and wrist to monitor the stationary cycling process; (**c**) the detected concentrations of Na+ and glucose, which were compared with the Ex situ calibration data. Adapted with permission from [15], Copyright (2016) Nature.

Homeostasis of metabolism parameters is critical for human health. Excessive variation of ionized metals, metabolites and acid-base balance can cause detrimental effects on the function of organs. In conventional approaches, blood samples are often collected and tested to evaluate the level of free ions and metabolites, which is suffering, tedious, invasive and time-consuming. To non-invasively and continuously monitor these biomarkers, many conformal bio-chemical sensors have been developed to measure the electrolytes and metabolites in excrements of body surface. Sweat is the most commonly used bio-fluid in non-invasive approaches because its electrolyte and metabolite are greatly related to blood plasma. The levels of Ca^2+^, Na^+^, K^+^, Cl^-^ and heavy metals in non-pretreated or filtered sweat have been evaluated by various wearable electrochemical sensors [15,89,90,91,92,93,94,95], and the well-designed selectivity capacitates one platform to simultaneously test different electrolytes, as seen in Figure 5 [15,90]. Meantime, the glucose in sweat can also be measured as an indictor to diagnose and treat diabetes with the help of efficient sweat collector and drug delivery component [14,96,97]. The lactic acid in sweat can be used to track an individual’s performance and examine the tissue oxygenation and pressure ischemia for elders [13]. Many of the abovementioned devices are constructed on a polymer substrate, and the impermeability and mechanical visibility may cause discomfort in long-term wearing. To further promote the compliance, Wang and Bandodkar proposed a series of tattoo-based electrochemical sensors, by which the sodium dynamics, glucose, pH, ammonium and alcohol in sweat had been detected [20,91,98,99,100]. Compared with the substrate-based devices, the tattoo-based sensors feature better mechanical compliance and excellent adhesion with skin. Except sweat, tear and saliva are also taken advantaged of as samples in metabolism assessment, but not as applicable as sweat in daily life [101,102,103,104]. The saliva-based health monitoring, often referred as salivaomics [105], have been greatly inspired by finding the bioinformatics in human saliva. As the key componens for salivaomics, wearable sensors are usually constructed in a mouthguard to promote their wearability. Different from sensing strips [106,107], the muothguard devices can continuously measure the saliva bioinformatics in vivo [108]. For example, the group of Prof. Wang has implemented the measurement of lactate and uric acid in saliva with the help of mouthguard devices [101,109]. The tested saliva can be directly and persistently loaded to the active electrodes in mouthguard, and the obtained signals can be transmitted by wireless electronics [109]. The measurement of metabolism parameters in tear is more difficult, and the soft contact lens (SCL) is regarded as one of the most desired candidate [110]. The group headed by Dr. Babak A. Parviz, a pioneer of SCL-based tear sensors, has developed several prototypes for monitoring the lactate and glucose in tear, which contains functional electrodes for target detection and antennas for communication and power transfer [111,112,113,114]. Meantime, the energy can also be provided by biofuel cells and its matched capacitor [115,116]. Some of the devices have been applied to rabbit eyes for the purpose of glucose monitoring, and the obtained results show a favorable correlation with blood glucose level [117]. However, the further investigation is still required in the reliability over time and temperature, high-efficiency transfer of signal/energy and air permeability.

In the devices for metabolism evaluation, pH, humidity and temperature sensors often work along with bio-chemical sensors. This arrangement can generate more comprehensive information about body condition, and compensate the measured results to get rid of the interference from these parameters [15,89,94,95,96,118,119].

## 3. Mechanisms, Materials and Fabrications

As previously mentioned, the health indicators can be detected as mechanical stimulations and concentrations of electrolytes or metabolites. The mechanical stimulations, including strain, pressure, force and vibration, can be transduced into electrical parameters by the wearable electromechanical sensors through several typical mechanisms, such as piezoresistivity, capacitance, and piezoelectricity, etc. The electrolytes and metabolites are usually evaluated by potentials of electrochemical sensors with the help of ionophores, reagents and electrodes. It is a key problem in constructing wearable sensing devices that the realization of flexibility and stretchability without any decline in testing capability and physical robustness. Meanwhile, the constructional material and fabrication strategy are critical factors for achieving the compatibility of mechanical and functional properties. In the following sections, we will first introduce the sensing mechanisms for different target stimulations; then, the constructional materials for sensor substrate and active element will be summarized; last, recent advances in device fabrication will be described.

### 3.1. Sensing Mechanisms

#### 3.1.1. Piezoresistivity

Piezoresistivity is the most popular transduction mechanism for the flexible/stretchable electromechanical sensors because of its simple configuration, straightforward operation and excellent sensitivity. The electrical resistance of piezoresistive sensors will exhibit variation under different mechanical excitations. Generally, the relative change of resistance (Δ*R/R*) can be written as:

(1) $$\frac{\Delta R}{R}=\frac{\Delta \rho }{\rho }+\left(1+2\;\nu \right)\varepsilon =G\;\varepsilon$$

where *ρ* is resistivity, *ν* is Poisson’s ratio, *ε* is applied strain, and *G* is gauge factor (GF). Herein, the GF, which represents measurement sensitivity, is determined by two terms: variations in geometry and variations in resistivity. The GF of commercial metallic strain gauges is derived from geometric variation with a typical value of ~2. The GF of semiconductors, like crystalline silicon for microsystems, can be up to about 200, which arises from the prominent variation in material resistivity. However, neither metallic strain gauge nor semiconductor-based piezoresistor are suitable for wearable flexible sensors. The former is restricted by its low GF, and the latter is impeded by its high rigidity and frangibility.

To enhance the GF and deformation range of piezoresistive wearable devices, many improved concepts have been proposed. These alternatives can be categorized into two modes: (1) varying the contract state of conductive elements and (2) tunneling effect. Benefiting from their inherent features and innovative structural engineering, the new modes have revealed typical characters in sensitivity and measurement range.

**Figure 6:**
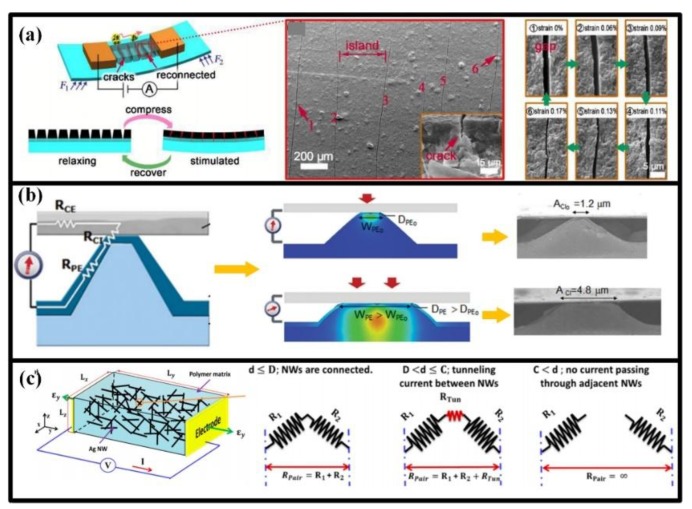
Available working concepts for piezoresistive wearable sensors: (**a**) micro-scale crack; (**b**) the connecting area between the active and electrode layers; (**c**) the connecting, disconnecting and tunneling effect between the adjacent active elements. Reproduced from [122] with permission of The Royal Society of Chemistry. Reproduced from [124] with permission of WILEY. Reprinted with permission from [35], Copyright (2014) American Chemical Society.

Micro-scale cracks in thin conductive film are excellent medium to implement the first mode. The motion of crack scarps can change the contract resistance between elementary units, and then trigger a great resistance variation to the film (shown in Figure 6a). The cracks can be produced by the evaporation of reagent in drying process, but their location and orientation cannot be well controlled [50]. To make the cracks more controllable, tension stress is applied and then offloaded to the dried active film, in which micro-scale cracks aligns along the tensing direction on a large scale [120,121,122]. Meantime, depositing the film onto rough surfaces, e.g., abrasive paper, can also generate high-quality sensing cracks [123]. The crack-based sensors have super high sensitivity in tiny deformations and can detect the very feeble strains in muscle motion and vital activity. Comparatively, the cracks cannot sustain large strains, and the measuring performance and device robustness will dramatically decline if the applied strain is out of affordable range.

Variation in the contact area between active and electrode layers can also result in an increase or decrease in overall contact resistance [124]. The varied gap usually generates from the rough surface in active and/or electrode layers, and the sandwich structure are often utilized with two electrode layers and at least one active layer. When stimulated by a pressure, the bulges in rough surface will be compressed, which will enlarge the contact area and then decrease the overall contact resistance, as shown in Figure 6b. When the load is removed, the bulges can rehabilitate and be ready for the next stimulation. The gap-based piezoresistive sensors will gain good sensitivity and response speed when the bulges are scaled to micro size. Moreover, the gap-based sensors can survive very large pressure and are suitable to plantar pressure measurement.

The varied connection between micro/nano conductive fillers in composites can also be utilized to construct piezoresistive sensors. Usually, the conductive fillers (particles, wires, tubes or flakes) are embedded into the insulated polymers to establish current percolating pathways. The resistance variation of such sensors mainly originates from the changes in inter-filler resistance, and the intra-filler resistance can be negligible. An example for this kind of strain sensor is the one based on pencil traces on paper, in which the graphite flakes may disconnect with or overlap each other. Though the contact variation between the graphite flakes has a huge impact on measurement sensitivity, the GF can be further enhanced by another working principle: tunneling effect, namely the electrons crossing two disconnected nanomaterials within a certain cut-off distance. For a pair of active elements, their connection state can be classified into three stages depending on their distance (Figure 6c) [35]. Firstly, they are fully connected with each other, and the resistance between them is dominated by the contact resistance, whose variation obeys the Ohm’s law. Then, they are separated by the applied strain, but the distance is still within a certain range. Herein, the tunneling resistance will become the largest component, which is determined by the Kirchhoff’s current law. At last, the two elements are further strained and completely disconnected with each other. Enhanced by the tunneling effect, the strain-resistance sensitivity can be up to several thousands, which is about an order of magnitude lager than the value in the situation with only contact variation.

Generally, most of the abovementioned sensors for motion detection are working based on piezoresistivity. Except that, piezoresistive sensors can also be used in measuring temperature, pH, analyst concentration and humidity [125].

#### 3.1.2. Capacitance

**Figure 7:**
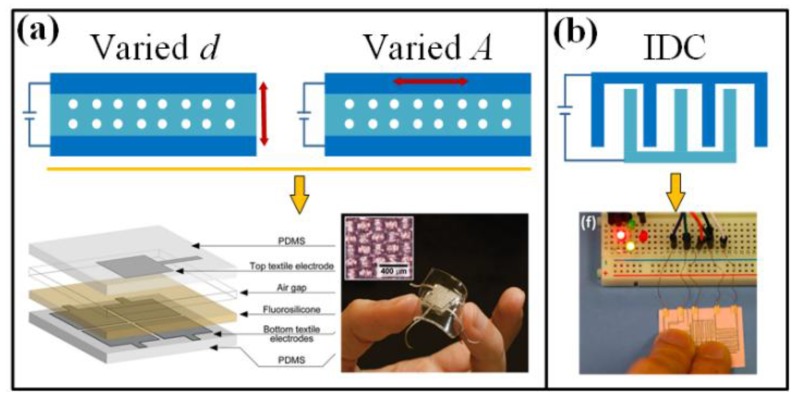
Available working concepts for capacitive wearable sensors, including varying *d*, *A* and IDC. Reproduced from [126] with permission of WILEY. Adapted with permission from [131], Copyright (2014) American Chemical Society.

For an ideal parallel plate capacitor, its capacitance *C* can be written as:

(2) $$C=\frac{A\;\varepsilon }{d}$$

where *A* is the area of one plate, *d* is the distance between the two plates, and *ε* is the permittivity of the dielectric material. The variation in dimension or permittivity, which might be induced by physical, chemical or biological stimuli, can be represented by the capacitance change. Same with piezoresistive ones, capacitive wearable sensors are also very suitable for detecting physical parameters. The applied load can cause a displacement to the plates, bring about a varied *A* and *d* and change the capacitance (Figure 7a). Herein, material compressibility is a key parameter that determines the deformation resistibility and directly correlates with measurement sensitivity. Using low modulus materials is a desired scheme for promoting sensitivity, and introducing microstructures into dielectric layer can also enhance the performance. Micro pyramidal bulges and voids are often utilized to embed air into dielectric to pursue highly sensitive and fast responding devices. Moreover, with the help of quadripartite textile electrode and air-fluorosilicone dielectric, a capacitive sensor for measuring three-axial forces are established with high flexibility, sensitivity, robustness and stability [126]. The parallel plate capacitive sensors have been used in monitoring finger and wrist motions [127,128,129], heart beating and breath [51,130]. Planar interdigitated capacitor (IDC) is another configuration used in flexible capacitive sensors (Figure 7b). When an object approaches, the electric field lines will pass through the new dielectric, which will change the sensor capacitance. This approach has been used in touch pads that compliance with complex surfaces [131,132,133] and stretchable instrumented insoles for gait detection [70].

#### 3.1.3. Piezoelectricity

Piezoelectricity is an electromechanical interaction in some non-centrosymmetric crystal structure materials, in which electric charges are generated when external mechanical stimuli are applied. This mechanism has been widely used in highly sensitive variation sensors with a cut-off frequency as low as 10^−2^ Hz. Inorganic piezoelectric materials, such as ZnO, GaN and PZT, have been widely investigated to construct flexible strain/pressure sensors by coating or embedding them to flexible polymers. Meantime, piezoelectric ZnO nanomaterials, especially nanowires, feature excellent piezoelectricity and have been used in nanosensors, piezotronic devices and nanogenerators. The group of Prof. Zhonglin Wang has have done outstanding work in this field, and the developed triboelectric mechanism can further extend the inorganic alternatives for piezoelectric wearable sensors. However, the utilization of inorganic materials has inherent disadvantages, such as high processing cost and high toxicity of lead. Thus, the utilization of piezoelectric polymer materials in flexible sensors has attracted increasing interests. Among them, PVDF is a promising candidate due to its mechanical flexibility, comparative piezoelectric coefficient, cost-effective processing technology, biocompatibility and chemical inertness.

#### 3.1.4. Others

**Figure 8:**
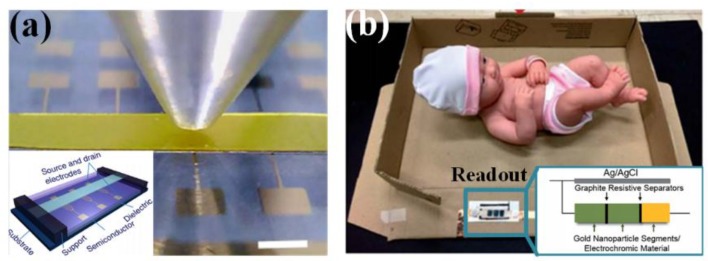
Other working mechanism for wearable sensors; (**a**) FET-based sensor; (**b**) visual sensing mechanism in the balance-in-a-box for infant birth weight determination. Adapted from [136] with permission of The Royal Society of Chemistry.

In addition to the previous categories, there are some other mechanisms for signal transduction. FET-based sensor can be implemented by incorporating sensitive resistor or capacitor into traditional transistor, which might be regarded as an expansion of piezoresistivity or capacitance mechanism (Figure 8a) [134]. However, benefiting from its perfect transduction and amplification functionality, potential of mass production and low power consumption, FET sensor has become an ideal candidate for flexible tactile sensing. Moreover, the FET array can reduce the crosstalk between sensing pixels, which usually happens in resistance or capacitance arrays. Hence, many studies on transistor array have been performed to develop large-scale and flexible artificial e-skin devices.

Optical transmitter/receiver pairs (e.g., LED and photodetector) are integrated into force-sensitive waveguides or optical fiber to detect the mechanical stimuli-induced modification in light wavelength or intensity. This method adopts optical parameters as the varied transduction medium, and establishes an electrical-interference-free approach for high-resolution touch screens and visual displays. Another visual sensing mechanism relies on the electroluminescence and electrochromatic phenomenon. The light luminance of powered ZnS composites can be changed by applied strains [135]; the color of Prussian blue/polyaniline segments varies along with the resistance of a weight-sensitive resistor, which has been used in a simple and low cost balance-in-a-box for infant birth weight determination (Figure 8b) [136].

In wireless sensing devices, the resonant frequency of sympathetic oscillation circuit can be drifted by mechanical stimuli. A flexible radio frequency identification (RFID) tag based on inductor–capacitor resonator has been made with a strain GF (frequency shift/strain) of 0.51 [137]. The proposed wearable tag can be potentially utilized in monitoring and protecting athletes for its capability of simultaneously identifying and sensing.

In the measurement of metabolism parameters, potentiometry is an available approach, and has been well investigated and widely used in analytical techniques. The working principle relies on the relationship between ion/reagent concentration and electrochemical potential of electrodes. A basic measurement unit consists of two working electrodes, a salt bridge and reference reagents to capture the target. The key to constructing a potentiometric sensor is finding proper reference reagent, which should provide excellent target selectivity and applicable operating potential. As mentioned in Section 2.3, this kind of sensors have been worn to detect metal irons, lactate, pH and glucose in human bio-fluids.

### 3.2. Construction Materials

The flexible/stretchable sensors are consisted of three basic components, including substrate, active element and electrode/interconnect. The organic materials have great mechanical flexibility and chemical stability, but very few of them reveal favorable active characters. Meanwhile, traditional inorganic electronic materials are sensitive to many stimuli, but not competent to mechanical compliance due to their rigidity and frangibility. Thus, the cooperation between different materials can be a solution to compacting high measuring performance, flexibility/stretchability and mechanical robustness in one device. The new approaches in material preparation, such as scaling down dimension and synthesizing composites, can be helpful in device development. The following parts will focus on the widely used materials and their participation in substrate, active element and electrode.

#### 3.2.1. Materials for Substrate

Substrate is the main source for flexibility and stretchability of wearable sensors, and directly determines the comfort level and long-term reliability. Organic materials, like polymers, silicone and rubbers, are met with great favor in substrate. Polydimethylsiloxane (PDMS), a commercial silicone elastomer with intrinsic high stretchability (up to 1000%), non-toxic, nonflammability, hydrophobicity and good processability, has been used in microfluidic, prosthesis and wearable sensors [138]. With the help of soft lithography, various microstructures can be fabricated onto PDMS films, making them more popular in highly sensitive, fast-response devices. The lithography mould can be a micromachined silicon wafer, a piece of silk and even a plant leaf. The fabricated patterns include grooves, pyramids, hemispheres, rods and random-distributed channels and their density and size can be easily adjusted. Ecoflex^®^ rubber is a newly developed, highly stretchable and skin safe silicone with better stretchability and lower modulus, which has been used in the sensors requiring more severe flexibility and stretchability [43,73]. Though cannot be stretched for its relatively high modulus (about 2~4 GPa), PET features good transparency (>85%), high creep resistance and excellent printability, and often appears as substrate film in wearable electrochemical sensors [15,89,90,93]. Polyimide (PI) film is another frequently-used substrate, which can maintain its flexibility, creep resistance and tensile strength under the condition of high temperature (up to 360 °C) and acids/alkalis [97]. Thus, PI film can participate in standard micromanufacturing process and then gains more diversity in designing and implementing wearable sensors. Except for the film form, polymer fiber and its textile can also be utilized as the structural core for depositing different active materials [36].

These abovementioned synthetic materials perform well as sensor substrate, but cannot be easily biodegraded, which will lead to environmental problems if the devices are widely used. Therefore, some natural materials are explored and developed to produce sensor substrate. Cellulose paper is inherently flexible, porous, inexpensive, recyclable, biodegradable and biocompatibility, and has been used in test strips for medical diagnosis. Recently, paper has shown a great promise in developing flexible devices, including strain sensors, transistors, supercapacitors, RC filters, RF antennas and plasma displays, etc. [31,123,125,133,139,140,141,142,143,144]. Moreover, the natural textiles, like silk and cotton, are also highly desirable substrate materials. Firstly, pony-size sensing chips can be integrated into these textiles to realize clothing-like sensing systems [145,146]. This scheme is easy to implement but suffers low-level integration. Then, fibers and textiles can be further modified by introducing conduction and sensitivity. The active yarns can be woven into textiles to realize the highly integrated multifunctional devices [147]. Meantime, active materials can also be absorbed into the porous fibers and textiles [30]. For example, a cotton sliver, which is dipped and dried repeatedly in silver nanowires (AgNWs) solution, features good electroconductibility (10^−5^~10^−4^ Ω/cm), and can be assembled into a high-performance pressure sensor with favorable sensitivity (3.4 kPa^−1^) and working stability (>5000 cycles) [148].

#### 3.2.2. Materials for Active Element

##### Carbon Materials

**Figure 9:**
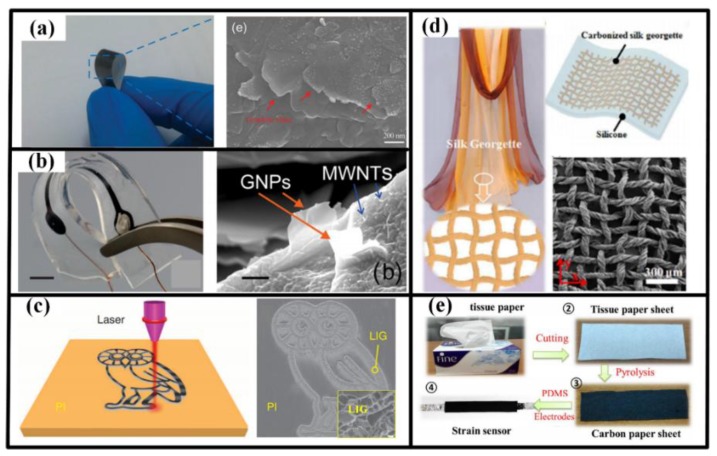
Carbon-based materials for sensing element: (**a**) graphene flakes from commercial pencil; (**b**) graphene flakes and MWCNTs compounded in substrate; (**c**) laser induced graphene on PI film; the carbonized silk georgette (**d**) and tissue paper (**e**). Reproduced from [139] with permission of WILEY. Reprinted from [138], with the permission of AIP Publishing. Adapted with permission from [149], Copyright (2014) Nature. Reproduced from [150] with permission of The Royal Society of Chemistry. Reprinted with permission from [152], Copyright (2016) American Chemical Society.

Different allotropes of carbon, such as graphite, carbon nanotube and graphene, have been widely used in fabricating wearable sensors (as shown in Figure 9). Graphite is an ancient carbon material, and recently returns to the academic sight with development of pencil-on-paper electronics [31,139]. In the drawing process, graphite flakes in pencil lead can be rubbed off by the physical friction between lead tip and porous cellulose paper, and deposited on paper surface. The drawn traces have been used as the resistor in RC filters and transistors. Moreover, the existence of structural edges in graphite flakes gives rise to a strain-induced resistance variation of pencil traces, making them suitable for strain gauges. By compressing the trace, more overlaps between graphite flakes are created, leading to a decrease to resistance; on the contrary, the tension strain separates more flakes and subsequently increases the resistance. The reported GF of the pencil-on-paper gauge is up to 536.61 (0.32% < ε < 0.62%), which is significantly enhanced by tunneling effect [139]. Meantime, the traces can also be used to construct planar IDC to detect finger touching and liquid pH. The functional ink based on graphite powder is another application form, and can be printed onto different substrates.

Carbon nanotube (CNT) is a one-dimensional allotrope of carbon, and features remarkable electrical conductivity and mechanical robustness. A single CNT is highly sensitive to strain (GF > 1000), but the related device is difficult to construct and use on a large scale. Therefore, CNT powder is often intermingled into polymer substrates to implement wearable devices, and its excellent conductivity plays an important role in sensor construction. CNT reagent can be deposited onto substrate to form electrodes in capacitive sensors, and dispersed into flexible/stretchable polymers to from piezoresistive composites or films. In piezoresistive composites, the network of CNTs presents an excellent strain sensing capacity, which is endowed by the strain-varied intertube tunneling resistance. It is noteworthy that the concentration of CNT should be controlled near the percolation threshold (PH) to maximize the GF. If the CNT loading is much lower than PH, many adjacent CNTs may be beyond their cut-off distance and the tunneling resistance will not work. Meanwhile, higher CNT loading can make more CNTs connect with each other, and the intertube resistance will be much smaller, which will significantly decline the GF. In piezoresistive films, the strain-induced variation of resistance can gain a ten-fold increment but its durability under cyclic loading is not favorable enough since an ultimate loss of conductivity will happen after hundreds of deformation cycles, which is induced by the rise of unrecoverable macro cracks and desquamations.

Graphene is the most promising active material for the development of flexible/stretchable sensors because of its outstanding electroconductibility (electron mobility of 2 × 10^5^ cm^2^·V·^−1^·S^−1^), excellent mechanical properties (in-plane stretchability of 25%, tensile strength of 125 GPa and Young’s modulus of 1 TPa), great thermal characteristic (thermal conductivity of 5300 W·m^−1^·K^−1^) and optical transmittance (>97%). Similar to CNT, graphene can be explored as electrode material for capacitors and filler for piezoresistive composites. Moreover, benefitting from its diverse fabrication methods, many unique devices are developed. For example, the laser scribed (LS) technique have been proved to be efficient in producing graphene layer onto various flexible substrates. The desired functional patterns are directly generated by irradiating graphite oxide (GO) films without any mask, template or transferring process. Additionally, the electronic properties of graphene layer can be easily adjusted in a wide range by varying laser power and irradiation time. The graphene woven fabric (GWF) is consisted of a large number of overlapping micro-ribbons and features a good trade-off between sensitivity and stretchability, making it suitable for wearable sensors. GWFs can be simply prepared by exposing the GO coated cotton bandage into ethanol flame, in which the pyrolyzation of cotton template and reduction of GO are synchronously conducted in tens of seconds. Fascinating stretchability (a tolerable strain up to 57%) and sensitivity (GF = 416 for 0% < ε < 40%, and GF = 3667 for 48% < ε < 57%) are simultaneously realized by encapsulating the obtained GWFs in natural rubber latex [72].

Inspired by the concept of low cost and environmental protection, many conventional materials are explored to synthesize carbon active materials. For instance, polyimide film has been directly scribed by laser to generate functional patterns with porous graphene, which can be applied as acoustic source and detector in artificial throat [12,149]. Carbonizing daily supplies also shows great potential in producing low-cost wearable sensors. For example, silk georgette has been carbonized in argon and hydrogen and then encapsulated by PMDS to construct a strain sensor, which possesses superiority in strain sensing range (ε > 100%), measurement sensitivity (GF = 29.7 for 0% < ε < 40%, and GF = 173.0 for 60% < ε < 100%), ultralow detection limit (ε = 0.01%), durability and stability (10,000 stretching cycles at 100% strain) and response time (<70 ms) [150]. Similar schedule is also applicable to tissue paper, cotton fabric and even wheat bran to realize sensors to monitor the vigorous and subtle motions of human body [74,151,152,153].

##### Metal Materials

Metal possesses excellent electrical conductivity and has been widely used in wearable sensors. Specific to active material, metal often appear in the following forms: (1) nano wires or particles; (2) flexible or stretchable configurations; (3) liquid state at room temperature. Nanowires (NWs) and nanoparticles (NPs) are often taken advantage of as fillers to prepare piezoresistive composites and conductive ink. For instance, AgNWs can be embedded into PDMS to build resistive-type sensors, but more sophisticated designs are needed here. The adhesion between AgNWs and polymers is not as strong as carbon materials, and permanent loss in AgNWs interconnection will happen when stimuli are loaded. The resistance will irreversibly increase after buckling and wrinkling if the AgNWs film is just simply coated on the surface of polymer. Therefore, AgNWs layer is often sandwiched between two substrate layers, forcing AgNWs to move back along their determined paths when the applied load is removed [35,154]. The conductive inks with metal NPs can be casted and annealled on the substrate surface to form the electrodes for capacitive sensors. The second working form of metal materials is on the basis of the strategy “structures that flexible and stretch”. Coiled buckled, serpentine and woven structures have been utilized to endow flexibility and stretchability to metals. Some good review works on the metal-based electronics with stretch structures have been conducted by Porf. John A. Rogers and Takao Someya, in which the available materials, ingenious configurations and practical applications are carefully described [155,156]. At last, liquid metal, like Ga and its alloys, maintains the liquid state at room temperature and is a nontoxic alternative to mercury [157,158,159]. Combined with microfluidic techniques, the liquid metals show a great capacity in constructing stretchable sensors. The applied mechanical stimulus will change the microchannel geometry, and then induce a significantly variation in the sectional area and length of liquid metal resistor, changing the electric resistance by as much as 50%. This approach can be directy used to measure pressure and strain, and assembled into a RFID tag or antenna to construct wireless sensing devices.

##### Polymers

Some organic materials possess favorable electro-properties and can participate in building active elements. An attractive feature of organic sensing materials is the mechanical similarity between them and many insulated substrate polymers. PEDOT-based polymers have been widely explored in sensing elements for their thermal stability, high transparency and tunable conductivity. Among them, PEDOT:PSS, a commercialized polymer, is one of the promising conductive organics due to its excellent solubility in water, which makes it compatible with many conventional processing techniques, like dipping-drying, spinning coating and inkjet printing. Meanwhile, the dried PEDOT:PSS film cannot sustain continuous bending and stretching cycles because of the intrinsic hard particles, which may trigger fissure and then decline in film conductivity. Therefore, the PEDOT:PSS ink is often printed and permeate into porous substrates, such as fabrics and cellulose paper, to promote the adhesion [36]. Encapsulated into substrate layers also helps a lot in upgrading film stability. PVDF is another active material with a number of attractive properties for piezoelectric wearable sensors. Thin and flexible sensors made of PVDF (and its copolymer PVDF-TrFE) have a wide range of applications in the field of healthcare measurements, including vital signals (heart rate and respiration) and plantar pressure distribution. Moreover, other organic materials such as PPy, P3HT and PANI also have been utilized to construct wearable sensors [160]. More recently, ionic liquid (IL), a kind of salt that keeping liquid state at room temperature, has attracted extensive attention in the study of electrochemical sensors, energy devices and transistors. Similar to liquid metals, IL can also be embedded in PDMS-based microchannels to construct strain sensors [49].

### 3.3. Fabrication Strategies 

Available fabrication strategies can be categorized into two groups: (1) compositing materials; (2) pattern transferring. However, the manufacture of wearable sensors often contains multiple processes, and many different techniques may involve. This part mainly focuses on the combination strategies for substrates and sensing elements, and some key processes for performance enhancement are also concerned.

Mixing different materials into a composite is the simplest fabricating approach. The active materials are doped into polymers by magnetically or ultrasonically stirring, and then the dried elastic composites can be prepared in bulk or film forms to fulfill application requirements. The mixed composites have complex electromechanical features that induced by the diversity of fillers and polymer substrates and the significant dependence on doping concentration and distribution state. Carbon black-silicone composite is a typical example that its characters significantly depend on the filler concentration. When the concentration of carbon black (CB) is low (0.08 wt %–0.09 wt %), the electrical resistance obviously increases with the applied uniaxial pressure; when the concentration comes to 0.1 wt %–0.13 wt %, the resistance appears a decrease-increase nonmonotone trend; then the electrical resistance decreases with the uniaxial pressure when concentration is larger than 0.14 wt % [161]. The filler dimensionality also has an impact on the electromechanical parameters of nanocomposites, which has been verified by the comparison of PH and GF between CB-PDMS and CNT-PDMS composites [162]. Encapsulation process can also yield sensitive composites, in which the active materials are sandwiched between substrates to form a substrate-sensitive composite-substrate configuration.

Pattern transferring is the most commonly used fabrication method for producing desired geometries in wearable sensors. The available techniques mainly include but not limited to micro-scale modeling, lithography, printing (e.g., screen printing, inkjet printing, 3D printing) and handwriting.

Micro-scale modeling is often utilized to prepare the microstructure in substrates, electrodes and sensing composites. The obtained components can be used to enhance the measurement sensitivity of piezoresistive and capacitive sensors by the concepts of gap configuration and microstructured dielectric. The modules can be micromachined wafers, silk fabrics and even plant leaves. In the modeling process, to-be-processed materials are poured onto the module and then peeled off after partially or completely drying. Overcoming the adhesion between processed material and module is a crux for this technique. Thus, necessary pretreatment and sophisticated geometric design are required to ensure the completion of peeling off process. The preparation of GWF is also derived from micro-scale modeling. Microstructured metal mesh is used as the module for depositing graphene, which will be etched away in the following steps.

Lithography is another pattern transferring method to realize diverse and ingenious geometries in flexible electronics. This process firstly deposits functional layer onto the substrate and then etches the undesired areas by reagent solutions with the help of photolithography. Since the high accuracy of photolithography and wet etching, the realized devices can obtain very sophisticated geometries and rich functionality. Utilizing this technique, Rogers’ group has constructed a series of epidermal electronics, including the abovementioned electronic system for measuring multiple signals in human motion [88], electronic ‘eyeball’ cameras [163], 3ω sensors for thermal characterization [164], multichannel antennas for brain function investigation [165], seamlessly integrated electronic system for feedback control [166], the epidermal coil for near-field communication [167], etc. Though limited by the available material and necessity of sophisticated equipment, this technology still opens a brilliant era for wearable electronics and gains great potential for commercialization.

Printing is an ancient technique and has played enormous roles in the spread of culture. Printing can simultaneously deposit and pattern many materials on various substrates without the need for sophisticated equipment and clean room. The wearable sensors can be printed with/without the help of masks, according to the specific implementation approach.

**Figure 10:**
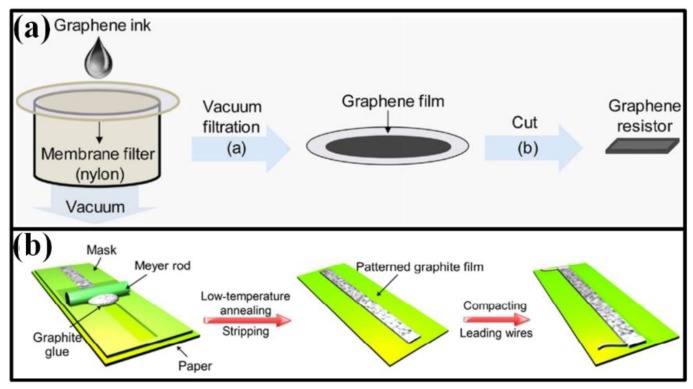
Available schemes for mask-needed printing: (**a**) vacuum filtration; (**b**) Mayer rod coating. Reprinted from [171], with the permission of Elsevier. Reproduced from [50] with permission of The Royal Society of Chemistry.

Screen printing is a typical mask-needed printing technique. Functional ink is forced into screen openings by fill blade or squeegee and transferred onto substrate surface. As the mask moves away, functional ink will remain on the substrate and form a patterned film. This technique has been widely used in manufacturing the working electrodes in electrochemical sensors and the sensing elements in electromechanical sensors [168,169,170]. Derived from screen printing, the ink can be forced onto substrate surface by vacuum filtration (Figure 10a) [120,171], spin/spray coating [40,51,130,172] and Mayer rod coating (Figure 10b) [50], in which the rheological properties of printed ink should be carefully designed.

For maskless printing, the to-be-transferred patterns should be predesigned and generated in a proper way. Stamping can be regarded as a simple maskless printing process. The embossed pattern can acquire functional ink and print it onto substrate [70,79,137,173]. This scheme works well in fabricating sensors with simple patterns, such as strip, rectangle and circle, which are often used to measure strain. Moreover, the CAD-based geometries can be directly printed onto substrate with the help of graphics software and printing equipment. Inkjet printing, propelling functional ink droplets onto paper, plastic or other substrates by a nozzle, represents an accurate, fast and reproducible film preparation technique and has been widely used in sensor development. The functional inks, including CNTs/graphene solution, conductive metals solution, polymer and liquid metal, should overcome the limitations on solubility, viscosity and surface tension, which to a certain extent increases the process cost. Another bottleneck in inkjet printing is the equipment. Professional inkjet printers are compatible with different functional inks and can automatically accomplish the repeated printing tasks, but their price is very high. Therefore, many dedicated facilities are developed to fulfill the special requirement from a certain ink. For example, a series of liquid metal printers have been developed by Liu et al. to easily and quickly manufacture personal electronics and circuits [174,175]. Moreover, the inkjet printing technique is also used in wet etching to fabricate the subtractive patterned platforms in organic electronic devices [176]. 3D printing is the best candidate for developing bizarre functional constructions and has gained great popularity in building flexible/stretchable sensors [177]. A three-layer sensor was produced in a single step by extruding viscoelastic carbon grease into elastomeric reservoir, which was originally constructed multiple steps in conventional scheme, including micro-molding, laminating and infilling [41]. More recently, a tactile sensor with an area of 3 × 3 mm^2^ and a height of 1.2 mm was constructed by a multimaterial, multiscale and multifunctional 3D printing approach. The cylinder-wall-structured sensing layer and woven electrode layers were sequentially printed from the bottom to top [178]. Benefiting from its small size and excellent sensitivity, the sensor was capable to detect and differentiate human motions.

Generally, lithography technique specializes in the fabrication of complex stretchable systems with high-precision dimension, delicate structure and rich functionality, but the processable materials and film thickness are limited, making them not suitable for the applications requiring substantial active materials. Meanwhile, the printing techniques, especially screen printing, can generate thick membranes from various materials, but its pattern resolution cannot meet the requirements from complex geometries. Thus, the hybrid fabrication process, combining printing and lithography, will certainly be a promising approach for manufacturing high-sensitive and well-patterned devices. Recently, Wang’s group demonstrated a series of high-performance stretchable “island–bridge” devices, in which screen printing was used to prepare the sensitive “island” units and lithography was employed for fabricating the serpentine interconnects [179]. The new hybrid fabrication strategy can lead to a variety of deterministic and versatile wearable devices, and be further improved by the tailored-made inks and tools for screen printing.

As previously mentioned, laser scribed (LS) technique also performs well in sensor manufacture. The laser beam moves in accordance with the pre-designed graphic, and the irradiated materials will be sensitized to sensing different stimuli. Laser reduced GO has appeared in many wearable sensors as the sensing element and shows great potential in high-performance artificial throat and skin [12,73]. Carbonating substrate material by one-step direct laser writing (DLW) has also been validated in manufacturing flexible electronics. Glassy and porous carbon structures have been produced from PI film via DLW. The lattice vibration induced by laser irradiation will generate extremely high localized temperature (>2500 °C), which can break the C-O, C=O and N-C bonds in PI and rearrange the left aromatic compounds into graphitic structure. It has been inferred that the aromatic and imide units are the key for carbon formation [149]. The DLW-based graphene possesses favorable electroconductibility, porousness and superhydrophilic wettability, and has been used in planar interdigitated microsupercapacitors [149], 3D conductive carbon circuits [180], microball lenses [181], wireless pressure sensor [182], flexible strain/tactile sensor [42,183] and gas sensor [184].

**Figure 11:**
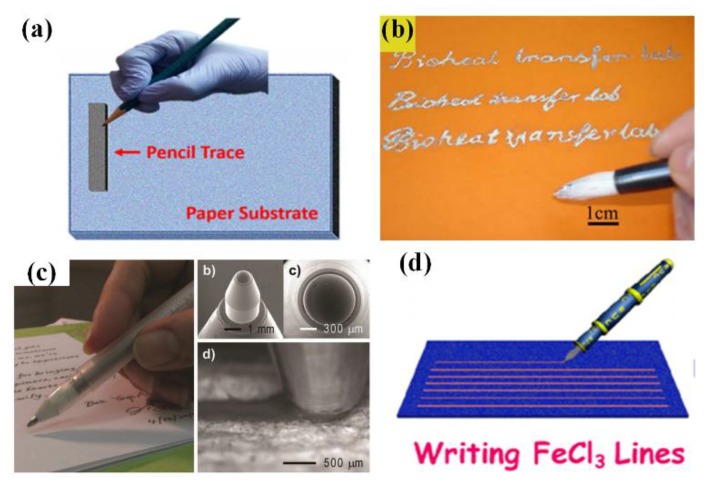
Directly writing electronics with different writing instruments: (**a**) Pencil; (**b**) Chinses brush pen; (**c**) Rollerball pen and (**d**) fountain pen. Reprinted from [133], with the permission of Elsevier. Reproduced from [189] with permission of WILEY. Reprinted with permission from [160], Copyright (2014) American Chemical Society.

Derived from day-to-day hand writing, directly drawing electronics with various instruments has recently become an alternative technique for fabricating low-cost, do-it-yourself sensors. This technique endows end-users the capability to design and realize sensors according to the “on-site, real-time” demands [32]. “Penciling it on”, as mentioned in previous section, has been proved to be a simple, rapid and solvent-free method for producing electronics (Figure 11a) [133]. Except the commercial pencil leads with determinate ingredients, many tailored leads are developed to fulfill the specific demands on “pencil traces”. The mixed powder of CNTs and selectors are compressed into a lead and then penciled onto a piece of paper to write an ammonia gas sensor. Moreover, Ag/AgCl modifiers are utilized to make the pencil trace suitable for reference electrodes in electrochemical and electrobiological sensors. The main defect of this technique locates at the demand on rough substrates to generate desquamation of pencil leads. Chinses brush pen, invented in Qin Dynasty of China (221–206 BC), is another available writing instrument for sensor fabrication. Similar to paintbrush, the low viscosity ink is firstly soaked into the animal hair bundle and then uniformly delivered onto the substrate by well-controlled handwriting manner. Benefiting from its excellent liquid manipulation, the brush pen can write electronics on various substrates, no need to consider rigidness and surface roughness. With Chinses brush pen and AuNWs/PANI ink, a high-performance strain-sensed tattoo can be utilized as HMI device to control robot arm [185]. Another typical application of this scheme is writing liquid metals. Liu’s group has written diversiform Gallium-based geometries on different substrates, including paper, wood, tape and human skin (Figure 11b) [82,128,186,187]. Accurately controlling the trace thickness and width may be a challenge for this method, though assistant mask and heater can partly solve these problems. Rollerball pen (Figure 11c) and fountain pen (Figure 11d) also work well in this field with functional inks loaded in their reservoirs [160,188,189]. With more sophisticated structures, rollerball pen and fountain pen can write with diverse inks (including metal inks, liquid metals and organic mixtures) to generate controllable geometries on many substrates, which has been used to realize strain gauge, glucose sensor, phenolic sensor and 3D antennas.

## 4. Performances and Challenges

High-performance and reliable wearable healthcare monitoring system requires flexible/stretchable sensors with various performances, including the basic ones (sensitivity, linearity, hysteresis, response time and durability) and specific ones (self-power, wireless communication, biocompatibility and biodegradability). Continuing progress in the enhancement and combination of these properties has been further exciting the wearable sensors to appear in more healthcare applications. However, some challenges still exit in the systematization, intellectualization and mass production of wearable healthcare devices.

### 4.1. Basic Performances

#### 4.1.1. Sensitivity and Linearity

Sensor sensitivity, namely the magnitude of electrical response to measured stimulus, is an important parameter for detecting subtle motions and scarce metabolites in human body. Measurement sensitivity can be affected by functional material, sensing mechanism and structural configuration. The materials with large piezoresistive or piezoelectric coefficient are desired, but employing the individual element of nanostructured materials is not a very favorable candidate. Herein, the enhancement approaches at macro scale are more practical. Tunneling effect and crack/gap structures in piezoresistive sensors have been proven to be effective in promoting sensitivity, and foamed/pyramidal elastomer dielectric also works well in capacitive sensors. It is noteworthy that most highly sensitive sensors can only maintain their superiority in a limited strain range, and the sensor with both high stretchability (ε ≥ 100%) and high sensitivity (GF ≥ 50) is still the focus of researches.

Linearity characterizes the proportional relationship between output signals and input stimulus, and excellent linearity can simplify the calibration and data processing process. However, simultaneously promoting sensitivity and linearity is also a great challenge. For example, piezoresistive sensors often exhibit varied GF in different strain ranges, which is induced by the nonlinear heterogeneous deformation. Meantime, capacitive sensors with microstructured dielectric also suffer the similar problem.

#### 4.1.2. Hysteresis and Response Time

Hysteresis and response time are key factors in evaluating sensor dynamical performance. Since most body motions are cyclic, a consistent sensing performance in loading and unloading is critical. Normally, capacitive sensors feature a lower hysteresis for its immediate responding to the variation of overlapped area. Meanwhile, piezoresistive devices are declined by the interactive motion between active filler and polymer substrate. The interfacial binding between filler and substrate is the critical parameter for hysteresis optimization. The interfacial slide under the weak binding hinders the fully recovery of filler position, and results in a high hysteresis behavior. Meanwhile, a weak adhesion is needed to avoid the friction-induced buckling and facture in fillers. Though the slide can be partially eliminated by low viscoelastic polymer substrate and improved configuration, optimizing hysteresis by novel material and structural engineering is still a large challenge. Response time illustrates the speed to achieve steady response to applied stimulus, and response delay exists in nearly all composite-based sensors because of the viscoelastic property of polymers. Relatively, piezoresistive device needs more time to reestablish percolation network in resistive composites, and thus has a larger response time than others. The utilization of lower modulus materials can further deteriorate the response speed of resistive sensors. However, the newly developed crack-based piezoresistive sensors feature a favorable response time (about 20 ms) because of the quick connection and disconnection of cracks upon the loading and offloading of stimuli. Thereby, innovative structural engineering can be an effective approach to improve the dynamical performance of wearable sensors.

#### 4.1.3. Durability

Durability determines the life of sustainably used devices. Firstly, cyclic stability represents sensor endurance to periodic loading-offloading cycles. The sensitive films coated on substrate usually suffers cyclic instable problem, in which buckling, facture and even stripping often appear after numerous cycles. For example, GWF film adopts a 24% drop in its peak value of relative resistance after about 1000 0% to 2% strain cycles, which is claimed to be caused by the weak interfacial interaction between GWF film and PDMS substrate [190]. This phenomenon also appears in crack-based piezoresistive sensors because of the additional propagation of sensing cracks [121,191]. On the contrary, the composite-based sensors usually feature excellent cyclic stability, and can maintain their characters after 10,000 cycles. For electrochemical sensors, the supply of reaction reagent directly determines sensor durability. Variation of potentiometry is mainly induced by reagent consumption, which can be optimized by improving operation form and reagent selection.

**Figure 12:**
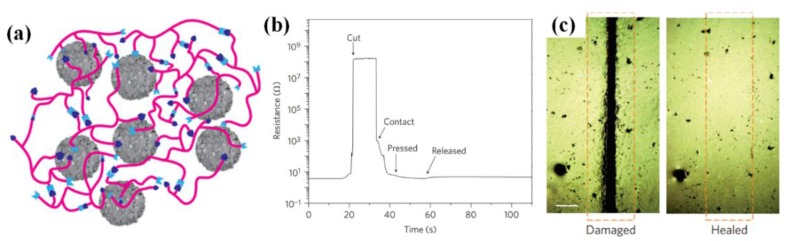
The tactile sensor with self-healing ability: (**a**) The interaction of oligomer chains with μNi particles; (**b**) The electrical healing process of resistance for 15 s healing time at room temperature; (**c**) Optical image of damaged sample and complete scar healing. Adapted with permission from [198], Copyright (2012) Nature.

Self-healing is another way to promoting durability. Though flexibility and stretchability have been realized, scratch and damage are still inevitable in daily usage, which will potentially decline the device functionality and service lifetime. Therefore, recovering the fundamental functions of sensors after damage, like the self-repair capability of human skin, has attracted intensive studies in recent years. The ideal approach is that the device can maintain its basic function after mechanical damage. Analogy with earthworms, the printable paper-based strain sensor can survive both lengthwise and transected cut, and preserve its high signal-to-noise ratio [50]. However, additional electrode adhesion and wire bonding are still needed to put the cut sensors into operation again. Herein, approaches that can heal the mechanical damage without complex process are more favorable. Embedding external healing agents and catalysts in capsule or vascular networks has been validated in the preparation of self-healing polymers [192]. The capsules will be broken by large strains and release healing agents into the crack region to trigger additional polymerization and link the separated molecules together again [193]. However, two problems exist here. Firstly, the extrinsic healing process can only recover the mechanical features, and the functional parameters are usually not well considered. To overcome this problem, Bandodkar et al. synthesized a kind of self-healing conductive ink to restore the mechanical and electrical contacts in printable electrochemical devices, whose broken capsules would release healing agent to locally dissolve the binder and then redistribute the filler particles [194]. Secondly, the depletion of agents restrains the persistent and multiple healing. The group of Prof. Zhenan Bao has made brilliant works in self-healing devices [18,195,196,197,198,199]. The first tactile sensor that can self-heal both functional and mechanical properties are constructed with the composite that combining organic supramolecular polymer and inorganic nanostructured μNi particles. The reestablishment of hydrogen bond between the damaged surface helps a lot in the self-healing process (Figure 12a) [198]. The Ni particles works as the conductive filer with a concentration under its PH to incorporate piezoresistivity, and at the same time as the motivator of hydrogen bond. In the proposed device, a reversion to 90% of its original electroconductivity can be achieved within 15 s after being ruptured (Figure 11b,c). Then, the magnet-based, gel-based, carbon-based and liquid metal-based self-healing mechanisms are successively developed, and their application field has been extended from the conductors and sensors to lithium-ion batteries, solar cells and energy storage devices [200,201,202]. More information can be found in the recent reviewing works by Zhang [203], Chen [204] and Amaral [205].

### 4.2. Specific Performances

#### 4.2.1. Biocompatibility and Biodegradation

Since many wearable sensors are intimately installed onto human skins, biocompatibility is, consequentially, a primary consideration. Generally speaking, active materials have higher risk than substrate materials. For example, it has been reported that injecting large quantities of CNTs into mice lungs could cause asbestos-like pathogenicity because of the small size and needle-like morphology of CNT [206]. Though this result is lately amended by a comparison between the lung inflammation caused by injections of well-dispersed single wall CNTs, asbestos and particulate matter collected from the air of Washington, DC [207], the potential toxicity of CNT remains a concern in medical application [208]. The cytotoxic effect of CNT and graphene have been verified by the biological experiment on neural phaeochromocytoma-derived PC12 cells, and the results are significantly concentration- and morphology-dependent [209]. Therefore, the future medical acceptance of nanostructured materials requires deeper understanding of immune response, along with the definition of exposure standards for different cases including inhalation, injection, ingestion, and skin contact [210]. Meantime, some organic active materials, such as PPy and PEDOT, have generally been found to be biocompatible and been utilized to monitor cell activities. The carbonized daily items, like cotton and tissue, also present great potentials in constructing biocompatible wearable sensors. As for substrate materials, favorable chemical/biological inertia and mechanical stability can ensure the biocompatibility, and a large number of applied substrates can meet these special requirements.

Another feature should be concerned in developing wearable sensors is biodegradation, which contains two aspects: absorbed by human organism and degraded by environmental microbes. First, resorbable materials, like silk derivatives, can be a candidate for degradable substrate [30]. For example, silk fibroin can offer an effective platform that possessing programmable dissolution and biodegradation rate, which can be implanted onto the surface of different organs to gather health indictors, such as electrocorticography and cardiac electrophysiology [211,212]. Then, environmental degradation is another concern. Many biodegraded materials, such as silk, cellulose paper, caramelized glucose, gelatin and starches, have been applied as substrates. Meanwhile, the biodegradation of active materials is a relatively complex problem. Recently, the pure magnesium (Mg) and its alloys have been developed as interconnects and implants in medical services, which feature outstanding biodegradation advantages over Fe-based and Zn-based ones [213,214].

#### 4.2.2. Wireless Communication

In a conventional monitoring system, the measured signals can be transported to the data collector by interconnect wires. However, troubles will arise if the similar scheme is directly used in daily health monitoring. The additional interconnect wires may break the life style of user, and the data collection and processing terminals can further burden the patients. Therefore, wireless communication has the operational necessity for high-performance wearable health monitoring system. In wireless communication, interconnect wires are replaced by wireless transceiver circuits, and processing module can be arranged in a remote control center, whose workable distance is determined by the transceiver. In general, integrating common wireless modules, such as ZigBee, Bluetooth and Wi-Fi, into wearable sensors is simple and easy to conduct. For example, the user-interface devices developed by Eom et al. used a XBee^®^ unit to send the acquired sensing signals [36]. However, this approach is poor in system integration, and inevitably enlarge the device size, which is not very desired in daily use. To overcome this shortcoming, specialized schemes are proposed to promote integration degree. In the wearable sensing array for in situ perspiration analysis, silicon integrated circuits for signal processing and wirelessly transporting are consolidated on a flexible circuit board together with sensing electrodes [15]. Benefiting from the tailored flexible board, the whole device can be worn as a wristband or headband. Another way to realize wireless communication is taking advantage of RF elements [137]. More recently, a novel wireless transmission based on triboelectric effect and electrostatic induction are presented, which shows remarkable capability of near-field wireless transmission and can further elevate the system integration [45].

#### 4.2.3. Self-Power

Power is the exciting element for the whole monitoring system. Enlarging the battery capacity and decreasing the power consumption are the two traditional ways to extend battery life. However, the usage of battery still causes incommodity in mobile wearable monitoring applications. Thus, self-power capability without the need for external power supply is particularly suitable for long-lasting wearable health monitoring system. Applicable methods for self-power in mobile system include scavenging energy from ambient environment and wirelessly receiving energy from power sources. First, the mechanical energy in human motions, such as walking, breathing and waving arms, can be harvested by piezoelectric and triboelectric nanogenerators. Then, light energy can in daily life can be harnessed by solar cells. Wireless coils, like RF antennas, have also been utilized as transmission medium to acquire energy. Meantime, the storage of adopted energy is also crucial. Mechanically flexible energy storage elements, mainly supercapacitor and lithium-ion battery, have been constructed and integrated into some monitoring systems. Fueled by the development of high-efficiency energy acquisition approach and ultra-low power consumption technique, significant progress can be achieved in self-power or even energy-free health monitoring systems.

### 4.3. Challenges

#### 4.3.1. Mass Production

Though different techniques and materials have been utilized to construct wearable sensors, the mass production of these devices still cannot be fully realized, due to the existing weakness in cost, fabrication efficiency and performance consistency. Most of active materials are synthesized by sophisticated processes, and their preparation cost may be a bottleneck. Fortunately, the yield and cost of CNT, graphene and other nanostructured wires/particles have been being improved, and the burdens from materials could be further released in the future. Meantime, the application of natural materials can provide more low-budget options and further diminish the economic burden. Then, the efficiency and performance consistency are not well ensured by current fabrication strategies. Firstly, some process parameters cannot be perfectly controlled, causing randomness in sensor performances. For example, the operation force in handwriting and stamping varies with people, and the obtained functional traces may feature different dimension and geometry; the cracks always appear randomly in the active films, resulting in different resistance and sensitivity. Printing fabrication techniques, like roll-to-roll printing and inkjet printing, have the potential of mass production, but practicable inks and substrates are limited.

#### 4.3.2. Multifunctional Sensing

It is expected that the monitoring system could provide a comprehensive information about health based on more indicators. However, just increasing equipped sensor is not very practicable because the system will become more complex and bring more disturbs to the wearer. Thus, extending the function of single device becomes a viable approach to measuring multiple parameters. For instance, Gao et al. had displayed a flexible sensor array for in situ perspiration analysis [15]. This device could simultaneously and selectively measure metabolites (glucose and lactate), electrolytes (sodium and potassium ions) and skin temperature, which enables the array to real-timely and comprehensively assess personal physiological state in a more personalized way. Moreover, the integration of ECG electrode, lactate sensor and heavy metal detector was also realized with the help of printing techniques. Generally, the multi-functionalization can be fulfilled by integrating multiple sensing units into one single pixel, and the crosstalk between these units should be extensively diminished by choosing proper pixel configuration and transduction principle. However, simultaneously measuring the mechanical and biological/chemical parameters is still a great challenge, which needs further supports from structure engineering and material technology.

#### 4.3.3. Systematization and Intellectualization

**Figure 13:**
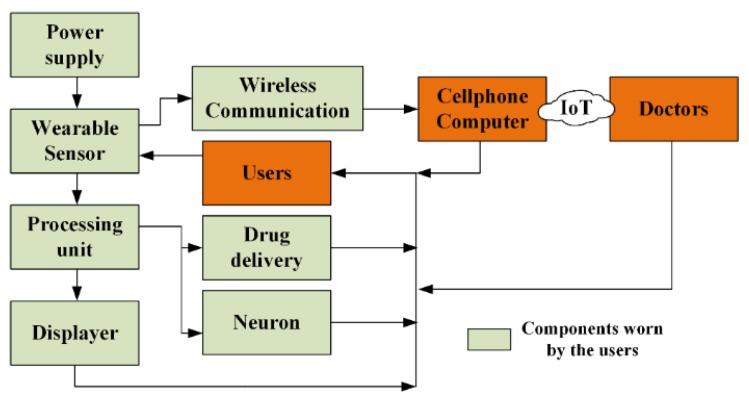
The ideal composition of wearable health monitoring system.

Systematization and intellectualization make wearable health monitoring system closer to market. More auxiliary components should be integrated into the system, such as unit for signal processing and displaying, feedback mechanism for therapy and element for power supply (Figure 13). Firstly, the system function can be further promoted if the closed-loop control of physiological parameters is introduced. For example, a feedback drug delivery module has been integrated into the sweat-based glucose monitoring system to timely control glycemic index [14,96,215]. Depending on the obtained glucose in sweat, an appropriate amount of metformin (or chlorpropamide) can be transdermally delivered by microneedles. Another systematization task focuses on the interconnection between monitoring devices and nervous system, which can be an important step toward the neural-integrated feedback system for disabled peoples to rebuild the sense of touch and muscle motion [216]. There are two levels of system intellectualization. Combining wearable system with personal computer or smartphone is the initial stage for intellectualization. With the help of appropriate applications (APPs) embed in smartphone, users can conveniently obtain sufficient information about their health [217]. Next generation of intelligent system may already contain the processing and displaying modules, which should be completely redesigned to meet the requirements on flexibility, minimization and integration. Some module prototypes, such as flexible screen and thin film transistor [218,219,220], have been developed, but further integration and minimization are still demanded.

## 5. Conclusions and Perspectives

In this review, we have highlighted the recent progress in flexible, stretchable sensors for wearable health monitoring as well as the considerable issues in their development. Detectable parameters for various health indicators have been measured by different wearable sensors. To construct high-performance sensors, piezoresistivity, capacitance and piezoelectricity mechanisms have been chosen and promoted by ingenious configurations. Meantime, the booming development of new materials (carbon, metal and polymer) and novel fabrication methods (compositing, printing and handwriting) leads to continuous advance in sensor performance. Many basic parameters of wearable sensors, such as sensitivity, linearity, durability and hysteresis, are getting closer to those of biological skin. Some unique and desired features, such as biocompatibility, biodegradability, wireless communication and self-power, have also been realized and boosted. Although significant progress has been made over the past decades, most of the wearable health monitoring systems are still at the prototype state, and great challenges remain in mass production, multi-functionalization, systematization and intellectualization.

Considering the existing challenges and the demands from ordinary users, additional works for the future researches of wearable sensors and health monitoring systems are predicted as following:

(1) Adoption by clinician and patient. Wearable device is an auxiliary appliance for health monitoring and diagnostic, and its compatibility with the existing medical system may be the first concerned issue. The compatibility firstly concerns the usability of the data obtained by wearable sensors. For example, the glucose level of sweat or tear can only be utilized to indicate diabetes after getting its exact relationship with blood glucose level. The evaluation of these wearable sensors by systematic medical studies is very necessary before the devices can be put into medical practice. Then, the adoption also requires a user-friendly design. Mechanical compliance of wearable sensors has been ensured by the excellent flexibility and stretchability, but few concerns are raised on the breathability. Human skin is also a metabolic organ with the function of perspiration and heat dissipation. Therefore, the attached devices should allow the heat, gas, and humidity to be repeatedly transported from one side of “artificial skin” to the other. Then, transparency is also desired to make the devices unnoticeable, so is the clothing- and tattoo-based schemes. Practicability is another concern, which demands conveniently wearing, simply operating and easily reading.

(2) Low-cost and high-efficient fabrication. As mentioned above, cost and efficiency is the main bottleneck in achieving mass production of high-performance sensors. Continuous investment in material preparation and fabrication process perfection can play a significant role in the construction of cost-effective, consistent sensors, whose effect can be further elevated by more ingenious structural designs.

(3) Higher integration. Herein, the integration involves multifunction sensing units and auxiliary components for power supply, communication and even signal processing and displaying. Multifunction sensing can acquire more health indicators with less sensing pixels, and the progress in flexible signal processing and displaying modules can make the systematization more realizable.

(4) Medical IoT. Medical IoT is important for preventive medicine architecture. The combination of IoT platform and wearable system can efficiently meet the requirement for self-health monitoring and preventive medicine raised by the projected dramatic increase in the number of elderly people.

In summary, wearable sensors and corresponding health monitoring system have been demonstrated as a feasible approach to address issues associated with real-time and high-efficient healthcare services. With the growing maturity of material science, fabrication technique, IC construction and structural engineering, the wearable health monitoring system will open up a new era for diagnosing, treating and preventing numerous diseases.

## Figures and Tables

**Table 1:** Detectable indicators in health monitoring.

Indicator	Position	Measured Parameter	Possible Application
Motion	Hand	Strain, Pressure	Rehabilitation, gesture identification
	Limb	Strain	Rehabilitation，sport training
	Foot	Pressure	Gait detection, sport training
	Throat	Strain	Phonation and deglutition detection
	Face	Strain	Expression identification
Skintemperature	The whole	Temperature	Wound healing, physiological status, Clinical diagnosis
Heart rate/ECG/pulse	Chest, wrist, neck, finger tip	Strain	Detection of Heart failure and cardiovascular disease
Respiration	Chest	Strain	Detection of cardiac arrest, apnea, emotional control
	Nostrils	Pressure, Humidity	
Metabolism	Sweating area, oral cavity, eyes	Constituent of biofluids	Evaluating the body status by the appearance & concentration of lactic acid, alcohol, electrolytes, pH
